# How Can the Health System Retain Women in HIV Treatment for a Lifetime? A Discrete Choice Experiment in Ethiopia and Mozambique

**DOI:** 10.1371/journal.pone.0160764

**Published:** 2016-08-23

**Authors:** Margaret E. Kruk, Patricia L. Riley, Anton M. Palma, Sweta Adhikari, Laurence Ahoua, Carlos Arnaldo, Dercio F. Belo, Serena Brusamento, Luisa I. G. Cumba, Eric J. Dziuban, Wafaa M. El-Sadr, Yoseph Gutema, Zelalem Habtamu, Thomas Heller, Aklilu Kidanu, Judite Langa, Epifanio Mahagaja, Carey F. McCarthy, Zenebe Melaku, Daniel Shodell, Fatima Tsiouris, Paul R. Young, Miriam Rabkin

**Affiliations:** 1 Department of Global Health and Population, Harvard TH Chan School of Public Health, Boston, Massachusetts, United States of America; 2 Division of Global HIV/AIDS, US Centers for Disease Control and Prevention, Atlanta, Georgia, United States of America; 3 Department of Health Policy and Management, Mailman School of Public Health, Columbia University, New York, New York, United States of America; 4 ICAP at Columbia University, Mailman School of Public Health, Columbia University, New York, New York, United States of America; 5 Center for Population and Health Research, Maputo, Mozambique; 6 Mozambique Ministry of Health, Maputo, Mozambique; 7 Oromio Regional Health Bureau, Ethiopia Ministry of Health, Addis Ababa, Ethiopia; 8 Centers for Disease Control and Prevention, Addis Ababa, Ethiopia; 9 Miz-Hasab Research Center, Addis Ababa, Ethiopia; 10 Centers for Disease Control and Prevention, Maputo, Mozambique; Banaras Hindu University, INDIA

## Abstract

**Introduction:**

Option B+, an approach that involves provision of antiretroviral therapy (ART) to all HIV-infected pregnant women for life, is the preferred strategy for prevention of mother to child transmission of HIV. Lifelong retention in care is essential to its success. We conducted a discrete choice experiment in Ethiopia and Mozambique to identify health system characteristics preferred by HIV-infected women to promote continuity of care.

**Methods:**

Women living with HIV and receiving care at hospitals in Oromia Region, Ethiopia and Zambézia Province, Mozambique were shown nine choice cards and asked to select one of two hypothetical health facilities, each with six varying characteristics related to the delivery of HIV services for long term treatment. Mixed logit models were used to estimate the influence of six health service attributes on choice of clinics.

**Results:**

2,033 women participated in the study (response rate 97.8% in Ethiopia and 94.7% in Mozambique). Among the various attributes of structure and content of lifelong ART services, the most important attributes identified in both countries were respectful provider attitude and ability to obtain non-HIV health services during HIV-related visits. Availability of counseling support services was also a driver of choice. Facility type, i.e., hospital versus health center, was substantially less important.

**Conclusions:**

Efforts to enhance retention in HIV care and treatment for pregnant women should focus on promoting respectful care by providers and integrating access to non-HIV health services in the same visit, as well as continuing to strengthen counseling.

## Introduction

The international community committed to eliminating the transmission of HIV from mother to child by 2015, with a target of 90% reduction of HIV infections among children, and to maintaining the health of mothers, with a target of 50% reduction in AIDS-related maternal deaths [1]. This ambitious plan was informed by the dramatic decrease in pediatric HIV and HIV-related maternal deaths in wealthy countries through universal HIV testing of pregnant women and use of antiretroviral treatment (ART) for eligible women. In the 21 African countries comprising nearly 90% of pregnant women living with HIV in the world, only 65% of HIV-infected pregnant women received antiretrovirals for prevention of mother-to-child-transmission (PMTCT) in 2012 [2].

Option B+, a new treatment approach that provides ART to all pregnant and breastfeeding HIV-infected women irrespective of disease stage or CD4+ cell count and continues such treatment for life, has been widely adopted as a means to protect women’s health and rapidly reduce vertical transmission and sexual transmission of HIV [3–5]. Because Option B+ calls for lifelong ART for all pregnant and breastfeeding HIV-infected women, it markedly increases the number of women eligible for lifelong ART, posing a potential health systems challenge.

Ethiopia and Mozambique have an estimated 760,000 and 1.6 million persons living with HIV, respectively [6, 7]. Rates of HIV transmission from mother to child are high; in 2012, 9,500 children were newly infected in Ethiopia and 14,000 were infected in Mozambique (Table 1) [8, 9]. At the same time, both countries suffer from severe health worker shortages, with health worker-population ratios of 0.28 per 1,000 population in Ethiopia and 0.37 per 1,000 in Mozambique, compared to 12.2 per 1,000 in the United States [6]. In implementing Option B+, Ethiopia and Mozambique have embarked on decentralizing ART services from hospitals to lower level clinics that provide antenatal and post-natal care with the goal of scaling up the necessary services [10, 11]. However, while Option B+ has increased access to ART for HIV-infected pregnant women, findings from Malawi indicated challenges in retention of the women in care, with 17% of pregnant women lost to follow-up 6 months after initiating ART. Compared to women starting ART for their own health (i.e., women with more advanced illness) Option B+ clients were five times more likely to never return after a first visit [12]. The authors suggest a number of reasons for lower retention, including that women at earlier stages of HIV disease perceive themselves as healthy and thus not in need of treatment, reluctance to disclose HIV status to family, and lack of support and counseling in busy clinics [12, 13]. A smaller qualitative study of Option B+ clients in South Africa identified work conflicts that interfered with keeping appointments, stigma, and negative treatment by staff as barriers to retention in care [14].

**Table 1:** Demographic and health profiles of study countries and regions.

National	Ethiopia	Year	Mozambique	Year
**Demographics**				
Population (thousands)	91,729	2012^1^	25,203	2012^1^
Percent urban	17	2012^1^	38	2012^1^
Literacy rate among adults aged ≥ 15 years (%)	47	2011^2^	44	2011^3^
**Health profile**				
Life expectancy (years)	65	2012^1^	53	2012^1^
HIV prevalence among adults aged 15 to 49 (%)	1.5	2012^1^	11.5	2009^3^
Under 5 mortality (per 1,000 live births)	68	2011^2^	97	2011^3^
Maternal mortality ratio (per 100,000 live births)	420	2013^1^	480	2013^1^
**Health system**				
Total health expenditure per capita (international $)	43.7	2011^1^	65.8	2012^1^
Midwives, nurses and doctors (per 1,000 population)	0.28	2012^1^	0.37	2008^1^
Antiretroviral therapy (ART)* coverage (%)	61	2012^1^	45	2010^1^
Antenatal care (ANC)^§^ coverage (≥1 visit; ≥4 visits) (%)	28; 12	2012^4^	89; 53	2012^4^
Births attended by a skilled health worker (%)	10	2011^2^	54	2011^1^
Contraceptive prevalence (%)	29	2011^1^	12	2011^1^
**Regional**	Oromia region (excl. Addis Ababa)		Zambézia province	
**Demographics**				
Population (thousands)	26,994	2007^5^	3,891	2007^3^
Percent urban	12	2007^5^	17	2007^3^
Literacy rate among adults aged ≥ 15 years (%)	39	2011^2^	28	2011^3^
**Health profile**				
HIV prevalence among adults aged 15 to 49 (%)	1.9	2011^2^	12.6	2011^3^
Under 5 mortality (per 1,000 live births)	112	2011^2^	142	2011^3^

^§^ Antenatal care (ANC) is defined as attended healthcare visits during pregnancy.

* Antiretroviral therapy (ART) consists of antiretroviral drugs to suppress HIV virus and stop progression of HIV disease. Percent ART coverage is calculated as: No. of individuals enrolled in ART / No. of individuals eligible for ART * 100

Sources:

^1^ World Health Organization. Global Health Observatory Data Repository: Ethiopia and Mozambique statistics summary. Geneva, Switzerland. 2013. UNAIDS. AIDSInfo Country Profiles. 2013.

^2^ Central Statistical Agency [Ethiopia], ICF International. Ethiopia Demographic and Health Survey 2011. Calverton, Maryland, USA: 2012.

^3^ Instituto Nacional de Estatistica. Panorama Sócio-Demográfico de Moçambique. Maputo, Mozambique: 2013.

^4^ UNFPA. State of the World's Midwifery, 2011: Country Profiles. 2012.

^5^ Central Statistical Agency [Ethiopia]. Ethiopia Population Census: Oromiya region profile: 2007.

These emerging concerns about retaining patients in lifelong treatment underscore the importance of identifying what HIV-infected women want from the health system and how services can best be organized to promote their retention in care and to optimize health outcomes. This is particularly urgent in resource-constrained settings where trade-offs in health system investments will inevitably be required. Discrete choice experiments (DCE) are a research tool for examining consumers’ stated preferences for health services. DCEs can be used to present hypothetical health care scenarios, each with different attributes (e.g., level of health facility, cost, availability of counseling services) to respondents who are then asked to select their preferred scenario. From these data the relative importance of each attribute can be estimated. One advantage of DCEs is the ability to test services and design changes ahead of their implementation [15, 16].

We conducted two discrete choice experiments (DCEs) in Ethiopia and Mozambique to identify the preferences of HIV-infected women of childbearing age for attributes of outpatient visits for ART in the context of lifelong care. The study was conducted in Ethiopia and Mozambique given the governments’ commitment to rapidly implement Option B+ and to learn whether preferences could be generalized across settings with different HIV prevalence. The findings from our study will inform policymakers in tailoring the provision of Option B+ to meet the needs of women and, in turn, increase uptake and retention in care.

## Methods

### Study setting and sample

Data for this study were collected using a cross-sectional survey that assessed health service utilization and preferences for HIV service delivery among HIV-infected women in the Oromia region of Ethiopia and the Zambézia province of Mozambique, two rural regions in which prior research has demonstrated that numerous well-known barriers to retention in HIV care and ANC persist [17–21]. We recruited study participants from health centers and hospitals supported by ICAP at Columbia University, an implementing partner for the President’s Emergency Plan for AIDS Relief (PEPFAR) supporting HIV-related programming in both countries. Facilities selected for the study were the four ICAP-supported facilities with the greatest volume of patients on ART in their respective regions, with between 1,500 to 3,000 women actively enrolled in ART at each site in 2013 [22]. Both Mozambique and Ethiopia introduced Option B+ in study clinics in June 2013, approximately one year prior to this study.

For this study, we recruited HIV-infected women aged 15 to 49 who were receiving care in selected clinics and who were either pregnant or indicated their intention to have children in the future. Participants were recruited from ART clinics, antenatal care (ANC) clinics, and in Mozambique, from clinics providing services for children born to HIV-infected women and their mothers as well as other high-risk groups. Given the marked difference in HIV prevalence between the two countries, we expected that 50% of our Mozambican sample and 10% of our Ethiopian sample would be pregnant women, consistent with prior trends we observed from clinic census data.

Researchers selected a systematic random sample by inviting women who were exiting their clinic appointments to participate in the study at regular time intervals during clinic operating hours. All eligible women were informed of the purpose of the study and their right to refuse participation. Interviews were performed after receipt of written consent from the participant or, in the case of minors, upon receipt of assent from the participant and consent from the guardian.

The study was approved by the ethics review boards at Columbia University Medical Center, the US Centers for Disease Control and Prevention, the National Research Ethics Review Committee at the Ethiopia Ministry of Science and Technology and the National Bioethics Committee for Health at the Mozambique Ministry of Health.

### DCE design

We conducted a literature review and held discussions with policymakers and clinical managers in both countries to determine an initial list of attributes for Option B+ services that were thought to be important to women and amenable to policy interventions. Where possible, similar attributes were selected to permit comparison between study findings in Mozambique and Ethiopia. Subsequently, we conducted four focus groups in each country with HIV-infected women in the reproductive age group in a subset of study clinics to narrow down the attribute list. Women were asked to choose HIV service attributes relevant to Option B+ most important to them and to suggest others not on the list. Based on these rankings, we identified two sets of attributes and levels for the DCE, one each for Mozambique and Ethiopia. The final attributes were: type of facility (hospital, clinic, and in Mozambique, mobile clinic), provider attitude, availability of integrated non-HIV health services (e.g. blood pressure testing, newborn care and family planning), availability of mother support groups (Ethiopia only), availability of counselor support, involvement of husband or family in care (Mozambique only), and total cost of the visit (Table 2). Cost of visit was included to permit estimation of willingness to pay. Though care and treatment for HIV infection is provided free of charge in both countries, women may bear other costs, such as transportation, food, and, if purchased privately, medications.

**Table 2:** Attributes and levels of health services used in discrete choice experiment.

*Ethiopia*		*Mozambique*	
Attribute	Level	Attribute	Level
Type of health facility	Hospital	Type of health facility	Hospital
	Health center		Health center
			Mobile clinic
Provider attitude	Health providers are respectful and welcoming	Provider attitude	Health providers are respectful and pleasant
	Health providers are not respectful and welcoming		Health providers are not respectful and pleasant
Non-HIV services	Non-HIV services available at the same consultation	Non-HIV services	Non-HIV services available at the same consultation
	Non-HIV services not available at the same consultation		Non-HIV services not available at the same consultation
Mother support groups	Mother support groups available	Husband/family involvement	Clinic involves husband or family in care
	Mother support groups not available		Clinic does not involve husband or family in care
Counselor support	Counselor to help you stay on treatment	Counselor support	Counselor to help you stay on treatment
	No counselor to help you stay on treatment		No counselor to help you stay on treatment
Cost*	0 Birr for each visit	Cost	0 MTn for each visit
	50 Birr for each visit		50 MTn for each visit
	100 Birr for each visit		100 MTn for each visit
	200 Birr for each visit		200 MTn for each visit
	300 Birr for each visit		400 MTn for each visit

*Note*. Introductory text: “In this section I will show you 9 cards. Each card describes two imaginary health facilities. These cards are not describing this facility or other facilities that you have used. Imagine that you are now pregnant and taking antiretroviral medicines (ART) to treat your HIV infection and to prevent your baby from getting HIV. You will need to take these medicines every day, throughout pregnancy and for the rest of your life. You will also need to visit a health facility ever 3 to 6 months for checkups and laboratory tests and to obtain medicines. You may need to come more frequently at the beginning. Please tell us which of the two facilities you would prefer to go to for your HIV care. Please keep in mind that you would need to return to this facility frequently so consider what services would be most important in helping you stay in treatment over many years. There are no right or wrong answers to these questions and remember we will not share your information with anyone. We are only interested in learning about what is important to you about health facilities.”

* 100 Ethiopian Birr = 5.12 USD and 100 Mozambican MTn = 3.20 USD; cost includes all expenses related to the care visit, including transportation and food.

The attributes and levels generated 160 possible alternatives in Ethiopia and 240 alternatives in Mozambique, which is too many for women to evaluate. Thus, we selected a fractional set of 16 alternatives or 8 choice sets using experimental design that maximized D-efficiency in Sawtooth Software, a software program for designing discrete choice experiments [23, 24]. To further enhance statistical efficiency and maximize study power we used five different versions of the DCE in each country. Each participant was presented with 9 choice cards, each showing two health facilities. One of the 9 cards was a “fixed choice” task that was used to assess the predictive validity of the model and not included in estimation of preference. After being read the introductory text and the attributes for each alternative, women were asked to select their preferred health facility to obtain Option B+ services and lifelong care for HIV infection. Each choice scenario was accompanied by a standardized script, which was read by the interviewer. Sample DCE cards are shown in S1 Fig.

### Non-DCE variables

The survey accompanying the DCE included questions about participants’ demographic and household characteristics, general and reproductive health history, HIV history, knowledge and stigma, and health system use and satisfaction with health services. We constructed a relative index of household wealth status using principal component analysis of a set of 20 questions on household assets [25].

### Survey fielding

The survey and consent forms were developed in English, translated to Portuguese in Mozambique, and Affan Oromo and Amharic in Ethiopia, then back-translated to English and pre-tested to ensure accuracy. The surveys were piloted, revised, and administered by trained interviewers. The interviews lasted 45 to 60 minutes and were conducted using hand-held electronic tablets with SurveyCTO software (Dobility, Inc., Boston, United States of America). Data were collected over a period of 8 weeks (16 April to 14 June 2014 in Ethiopia, and 8 April to 6 June 2014 in Mozambique).

### Statistical analysis

Data were cleaned (typographical errors corrected, variables recoded as necessary) and transferred to Stata v.12 (StataCorp LP, College Station, TX, USA). We calculated descriptive statistics for survey variables. We then fit mixed logit regression models with DCE attributes and levels as explanatory variables using Stata’s mixlogit command to estimate relative utility of each DCE attribute. Mixed logit models are commonly used to analyze discrete choice data as they account for taste heterogeneity by allowing attribute coefficients to vary across respondents; they also control for intra-individual correlations due to repeated responses [26]. Mathematically, mixed logit differs from the standard logit model in that the researcher does not know the value of *β*_*n*_ or *ε*_*in*_. For this reason, the solution of the equation requires that *L*_*ni*_ be integrated over all possible values of *β* weighted by the density distribution—usually a standard normal. The unconditional probability of the observed sequence of choices for a given choice set *t* is given by $P_{nit}(\theta )\;=\;\int L_{nit}(\beta )f(\beta |\theta )d\beta$.

Probabilities were estimated with a simulated maximum likelihood estimator. The output of a mixed logit model includes mean attribute utilities and standard deviations of the random coefficients, the latter reflecting the degree of preference heterogeneity among respondents.

We assessed the predictive validity of these base mixed logit models by comparing the model-predicted choice of health facility in the fixed choice task with the respondent’s actual choice. Lastly, we fit additional models with interaction terms between each attribute and pregnancy status to explore whether pregnant respondents, respondents not on ART, and women who were potentially eligible for Option B+, and younger women (under 25) had different preferences. We defined potential Option B+ clients as women who were pregnant or breastfeeding and not on ART or were on ART only during this or past pregnancies (i.e., pregnant or breastfeeding women not on ART for their own health). In all models, as per standard practice, costs of the visit were specified as fixed to ensure ease of interpretation of willingness to pay, and all other variables were specified as random [27].

## Results

### Sample characteristics

Of 1,036 women eligible to participate in Ethiopia, 1,013 participated in the survey, for a response rate of 97.8%. In Mozambique, 1,020 of 1,077 eligible women participated in the survey, for a response rate of 94.7%. Demographic and health characteristics of the respondents are summarized in Table 3. The mean age was 30.5 years in Ethiopia and 25.3 years in Mozambique. Most respondents were on ART at the time of the interview (94.8% in Ethiopia and 85.7% in Mozambique). Women known to be pregnant at the time of the interview comprised 11.5% (n = 117) of the sample from Ethiopia and 48.9% (n = 499) of the sample from Mozambique.

**Table 3:** Characteristics of study population by country, Ethiopia and Mozambique, 2014.

	Ethiopia (n = 1,013)	Mozambique (n = 1,020)
	n (%)	n (%)
Demographic		
Age, mean (SD)	30.5 (4.9)	25.3 (5.4)
Respondent is head of household	222 (24)	165 (16.2)
Currently married or living with partner	725 (72)	754 (73.9)
No. of children, mean (SD)	1.6 (1.2)	2.5 (1.7)
Educational attainment:		
*No education*	235 (23)	120 (12)
*Any primary*	379 (38)	467 (46)
*Any secondary*	395 (39)	433 (43)
Literate^1^	723 (71)	710 (70)
Primary occupation as farmer or homemaker^2^	494 (49)	712 (70)
Resides in village (vs. town)	117 (12)	640 (63)
Household assets		
Household has electricity	931 (92)	581 (57)
Household has radio	824 (81)	521 (51)
Household has television	713 (70)	519 (51)
Household has a mobile phone	931 (92)	747 (73)
Wealth quintiles		
Highest	202 (20.0)	202 (20.0)
Fourth	201 (20.0)	202 (20.0)
Middle	203 (20.1)	202 (20.0)
Second	202 (20.0)	202 (20.0)
Lowest	203 (20.1)	203 (20.1)
Pregnancy and ART status		
Currently pregnant	117 (12)	499 (49)
Not on ART now^3^	53 (5)	146 (14)
Potential Option B+ client^4^	55 (5)	370 (36)

Note: Data are no. (%) of subjects, unless otherwise indicated. For some rows, denominators differ from country totals owing to missing data.

^1^ Has completed primary school or was able to read all or part of a sentence in the national language (Amharic or Affan Oromo in Ethiopia; Portuguese in Mozambique)

^2^ Includes homemakers, farmers and house cleaners.

^3^ Women not on ART now include women who initiated ART at the current visit. This includes pregnant and non-pregnant women, i.e., current and future potential Option B+ clients.

^4^ Potential Option B+ clients include women who are pregnant or breastfeeding and not on ART orare on ART but have only taken ART during pregnancies. These women are not on ART for their health.

Among our sample, 55 (5.4%) women in Ethiopia and 370 (36.2%) women in Mozambique could be characterized as currently eligible for Option B+—i.e., they were pregnant/breastfeeding and not currently on ART or had been on ART only during pregnancies. The length of diagnosed HIV infection (calculated as the difference between the self-reported date of first HIV diagnosis and date of interview) among these women was shorter than for women on treatment for their own health (mean number of months since HIV diagnosis in Ethiopia: 51.2 vs. 64.9; Mozambique: 6.6 vs. 34.6). The majority of Option B+ clients (98.2% in Ethiopia and 100.0% in Mozambique) were recruited from antenatal clinics. Their average age was 28.0 years in Ethiopia and 24.2 years in Mozambique.

### DCE utility estimates

Utility estimates for DCE attributes are shown in Table 4. All of the attribute levels were significantly associated with health facility choice at α = 0.05. In the main effects model, five of the attributes–provider type, provider attitude, availability of non-HIV services in the same consultation, availability of counselor, availability of mother support groups (Ethiopia only), involvement of husband or family in care (Mozambique only)–had positive mean coefficients representing positive preferences for these attributes. As expected, increasing cost had an aversive effect on preference, indicated by the negative coefficient. Our models correctly predicted a high proportion of alternatives selected in the fixed choice cards: 86.7% in Ethiopia and 91.1% in Mozambique; confirming that the models included important characteristics and were thus well-specified.

**Table 4:** Results of mixed logit regression models.

Ethiopia					Mozambique				
Attribute	Mean^1^	SE^2^	SD	SE	Attribute	Mean^1^	SE^2^	SD	SE
Non-HIV services available at the same consultation	2.31	0.12**	2.08	0.12**	Non-HIV services available at the same consultation	1.06	0.07**	1.27	0.08**
Providers are respectful and welcoming	1.78	0.09**	1.51	0.09**	Providers are respectful and pleasant	1.61	0.08**	1.38	0.08**
Mother support groups available	1.06	0.06**	0.66	0.10**	Providers involve husband/family in care	0.67	0.05**	0.91	0.08**
Counseling services available	0.94	0.07**	-0.57	0.14**	Counseling services available	0.57	0.05**	0.76	0.06**
Hospital (vs. health center)	0.37	0.06**	0.88	0.09**	Health center (vs. mobile clinic)	0.16	0.06**	-0.25	0.18
					Hospital (vs. mobile clinic)	0.15	0.05**	0.38	0.14**
Cost (continuous in 100 Birr)^3^	-0.46	0.03**			Cost (continuous in 100 MTn)^3^	-0.19	0.02**		
Model diagnostics					Model diagnostics				
Number of respondents	1,013				Number of respondents	1,020			
Number of observations	16,192				Number of observations	16,156			
Log-likelihood	-3565.6				Log-likelihood	-4183.7			
Likelihood ratio χ2	939.92				Likelihood ratio χ2	635.79			

^1^ Mean β coefficients show estimated utility of each attribute, where positive coefficients indicate positive preference.

^2^ **p < .01

^3^ Currency equivalents in USD are 100 Ethiopian Birr = 5.12 USD and 100 Mozambican MTn = 3.20 USD, using period average exchange rates for the dates of data collection, extracted from OANDA.com (Ethiopia: 16 Apr 2014 to 12 Jun 2014; Mozambique: 8 Apr 2014 to 23 May 2014).

The two most important attributes for women in both countries were respectful provider attitude and availability of non-HIV services (such as blood pressure measurement, newborn care and family planning) in the same consultation. To illustrate the magnitude of utility gain for women associated with these attributes, good provider attitude was twice as important to women compared to availability of counseling services when choosing a health facility for HIV care in Ethiopia and three times as important as counseling in Mozambique (Ethiopia: β = 1.781 vs. 0.936; Mozambique: β = 1.606 vs. 0.573). Availability of non-HIV services in the same consultation was the most valued attribute in Ethiopia and was also two to three times as important as counseling services, though in different order of importance between the two countries (Ethiopia: β = 2.312 vs. 0.936; Mozambique: β = 1.058 vs. 0.573). By contrast the facility level was consistently the least important attribute (Ethiopia: β = 0.368 for hospital vs. health center; Mozambique: β = 0.163 and β = 0.150 for health center and hospital vs. mobile clinic, respectively). This means that women place relatively less emphasis on type of health facility where they obtain HIV care, holding all other attributes constant.

Supplementary analyses containing results from models that included interaction terms between service attributes and pregnancy status, ART status, current potential B+ clients, and young age (under 25 years) are presented in S1–S4 Tables. Pregnant women in Ethiopia valued mother support groups more than did non-pregnant women, and in Mozambique they valued respectful providers more than did non-pregnant women. In Mozambique the involvement of husband or family was less important to pregnant women than to their non-pregnant counterparts. Analyses with interaction terms for ART status indicate that in Ethiopia there were no differences in preferences between women currently on ART vs. not currently on ART. In Mozambique, women on ART preferred availability of non-HIV services more than did women not on ART, and they were also more averse to increased cost. Women identified as current potential B+ clients (pregnant or breastfeeding and not on ART or on ART during pregnancies only) were compared to other women. In Ethiopia, these women valued mother support groups and receiving care at hospitals vs. health centers more than did non-B+ clients. In Mozambique, B+ clients preferred availability of non-HIV services and respectful providers more than did non-B+ clients. Lastly, analysis that included interaction terms between service attributes and young age (under 25 years) show no differences between younger women and older women (see S1–S4 Tables). Analysis of dominant attributes, i.e., when respondents always selected alternatives with a specific level of one attribute, indicated that up to one-fourth of respondents had dominant preferences for the top attributes of good provider attitude and availability of non-HIV services. We elected to retain these respondents in the analysis as a dominant or non-trading response pattern may be consistent with random utility theory and deleting these responses may thus remove valid information about preferences. Removing respondents with dominant preferences can also induce selection bias, reducing generalizability to the broader population, and reduce statistical efficiency of the model [28].

## Discussion

This is the first DCE to assess women’s preferences for the structure and content of lifelong antiretroviral treatment in the context of Option B+ scale up in sub-Saharan Africa. We found that HIV-infected pregnant women and women desiring a future pregnancy in Ethiopia and Mozambique placed greatest value on respectful providers and the ability to obtain non-HIV services in their clinic visits for HIV treatment. To illustrate the magnitude of preference, these two service attributes were approximately twice as important to Ethiopian women as the availability of mother support groups and counseling services. In Mozambique, women valued respectful providers more than twice as much as providers who involved husband/family in care and valued the availability of non-HIV services in the same visit twice as much as obtaining counseling services.

These findings suggest that women’s choice of clinics for lifelong ART in the context of the Option B+ strategy is influenced by desire for respectful care and convenient access to non-HIV services alongside HIV services—characteristics that are not specific to HIV care but rather reflect an effectively functioning, patient-centered health system. Additional analysis showed that the utility of respectful providers was even greater for pregnant women in Mozambique than non-pregnant respondents.

The high value for provider respect is consistent with DCE studies of obstetric care: women in Tanzania and Ethiopia were highly influenced by having a respectful provider in deciding between hypothetical facilities for delivery [29, 30]. Consistent with this, researchers found that harsh treatment from nurses was identified as a barrier to retention in care by women enrolled in Option B+ in South Africa [14]. Researchers in Malawi observed that provision of Option B+ in large clinics was associated with greater loss to follow-up than in smaller clinics, hypothesizing that this may have been in part due to overworked staff who had less time for clients [12].

Women in both countries in our study highly valued the ability to obtain needed non-HIV related services in their HIV clinic visit. This suggests that women would like to improve the efficiency of their encounters with the health system, a particularly trenchant concern given the need to return to clinic many times for HIV care and treatment [31]. This highlights the importance of better organizing outpatient care to permit access to more than one service and is consistent with recent PEPFAR priorities that promote integration, sustainability and coordination between countries [32]. Situating Option B+ services in maternal and child health, which is the model in Mozambique and other countries, may facilitate service integration and potentially promote retention in care. While much has been written on the benefits and costs of integration of HIV services with other health care, the finding that clients with HIV might choose their facility based on convenient access to a range of services is novel and of substantial policy relevance [33–35].

Availability of counseling, mother support groups (Ethiopia), and counseling and providers who involved the husband or family in care (Mozambique) were positively associated with clinic preference. Pregnant women in Ethiopia had a stronger preference for mother support groups than non-pregnant respondents. The value placed on peer support is consistent with the work of Assefa et al. who found that community involvement in care was an important driver of high retention in care after service decentralization in Ethiopia [36].

Pregnant respondents in Mozambique indicated a lower preference for provider efforts to involve the husband and family compared to non-pregnant women. Reluctance to disclose HIV status is likely influenced by prevailing stigma [37, 38]. However, more work is required to understand how this might differ for pregnant versus non-pregnant women and how clinicians can support disclosure in settings where stigma remains a reality. Mozambique is currently considering the introduction of peer support groups to promote adherence and reduce stigma. Our findings from Ethiopia buttress this as mother support groups were valued by respondents there.

Study participants in both countries indicated that the type of facility—hospital versus health center (or mobile clinic in Mozambique) was much less important to them than other factors. This is reassuring for policymakers as decentralization of HIV care to health centers and clinics is proceeding rapidly in both countries. The important caveat is that the indifference to service setting is conditional on receiving other valued attributes on the list: respectful care, access to non-HIV services, counseling, etc. Thus facilities selected for Option B+ services—no matter what their level—need to be well-functioning and include a basic package of non-HIV services [39].

The study had several strengths. This is the first discrete choice experiment exploring preferences for lifelong receipt of HIV treatment. The study involved a large number of HIV-infected women and included both current and potentially future clients for Option B+. Finally, the robustness tests that compared model-predicted with actual choices of DCE facilities suggested that the experiment was well specified, capturing important drivers of preference. This study had also several limitations. First, all stated preference studies indicate respondents’ preferences for hypothetical choices; validating this work with revealed preference data from a policy experiment is important to confirm the findings. The non-DCE variables were self-reported and are subject to recall bias. Second, the study was done in a limited number of facilities in Ethiopia and Mozambique and the results cannot be extrapolated to other populations. However, the concordance of preferences across the two countries, particularly the strong preference for the top two attributes, suggests our findings may be applicable to demographically and geographically comparable populations facing similar health systems challenges. Third, interviews were only conducted with women at health facilities, and the vast majority of participants were already receiving ART. Therefore we could not capture HIV care preferences of infected women who are not yet diagnosed or have discontinued care. Finally, the sample of pregnant women who met criteria for Option B+ was relatively small in Ethiopia, limiting inference.

An important implication of our findings is that health systems need to make structural changes such as improved staffing and service availability in order to enhance lifetime retention in HIV care and improve the quality of interpersonal care and access to a range of health services [40]. The emphasis on service availability and convenience is consistent with the fact that women with HIV perceive their HIV infection as one of several health needs that might require medical care.

Another central findings was the high value placed by women on respectful treatment. The demand for better interpersonal quality of care may reflect that women experience brusque and disrespectful treatment by providers in resource-constrained health systems [40, 41]. The need for effective communication and more empathetic treatment has been amply demonstrated in HIV care although the evidence on what works to ensure respectful treatment by providers is sparser [42, 43]. Research on underlying causes of disrespectful treatment and on interventions that address this is urgently needed. Potential approaches that have been documented in the literature include increased staffing—which reduces provider perceptions of feeling overburdened—thereby improving their ability to deliver care, pre-service and in-service training on respectful care, supportive supervision, task-shifting of services to other cadres to reduce workloads on clinicians, building interpersonal skills and efforts to improve facilities’ accountability to their communities, such as community-facility committees and community charters [44, 45]. Our findings show that it is essential for policymakers and providers in HIV care to embrace the respectful care agenda now being promoted in other areas of health [31].

Option B+ offers an important opportunity for enhancing health outcomes for HIV-infected pregnant women and their infants. However, none of these benefits can be achieved without high rates of retention of the women in care. Our study findings indicate that in order to create people-centered health systems and to ensure that the services provided are services that people will use, policymakers must know what users value and shape the health systems in a manner to address those preferences [46, 47].

## Supporting Information

S1 Dataset B+ discrete choice experiment dataset. (DTA)

S1 Fig Sample DCE cards. (DOCX)

S1 File B+ discrete choice experiment dataset codebook. (XLSX)

S1 Table Results of mixed logit regression models with interaction terms with pregnancy status (pregnant vs. non-pregnant). (DOCX)

S2 Table Results of mixed logit regression models with interaction terms with ART status (Not currently on ART vs. on ART). (DOCX)

S3 Table Results of mixed logit regression models with interaction terms with current potential B+ clients (women pregnant or breastfeeding and not on ART or on ART during pregnancies only (pMTCT) vs. others). (DOCX)

S4 Table Results of mixed logit regression models with interaction terms with age less than 25 (<25 vs. 25+ years old). (DOCX)
