# Supplementary figures and images for: How Can the Health System Retain Women in HIV Treatment for a Lifetime? A Discrete Choice Experiment in Ethiopia and Mozambique
Source: PLoS One. 2016 Aug 23;11(8):e0160764. doi: 10.1371/journal.pone.0160764 (PMC4994936; doi:10.1371/journal.pone.0160764)

S1 Fig. Sample DCE cards


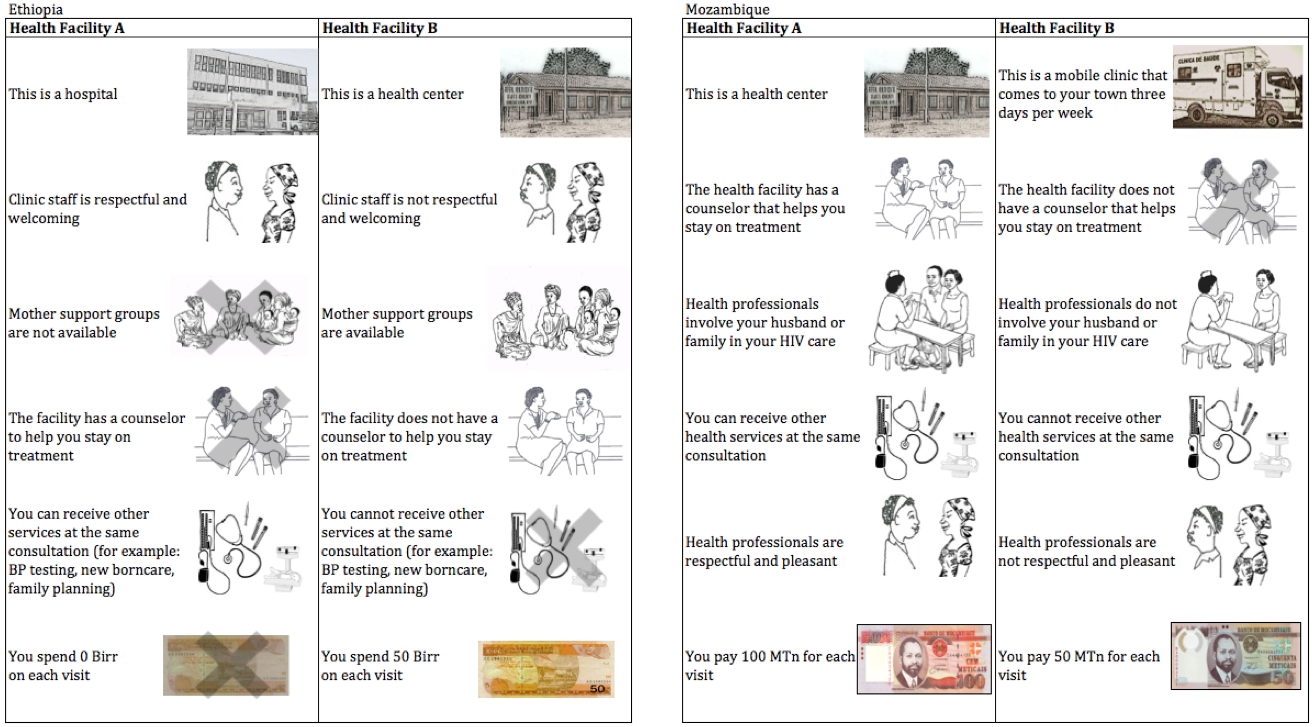

Supplement: S1 Fig — (DOCX) [file pone.0160764.s002.docx]
