# Supplementary material for: How Can the Health System Retain Women in HIV Treatment for a Lifetime? A Discrete Choice Experiment in Ethiopia and Mozambique
Source: PLoS One. 2016 Aug 23;11(8):e0160764. doi: 10.1371/journal.pone.0160764 (PMC4994936; doi:10.1371/journal.pone.0160764)
Supplement: S1 Table — (DOCX) [file pone.0160764.s004.docx]

S1 Table. Results of mixed logit regression models with interaction terms with pregnancy status (pregnant vs. non-pregnant)

| Ethiopia | | | | |  | Mozambique | | | | | | | | | | | |
| --- | --- | --- | --- | --- | --- | --- | --- | --- | --- | --- | --- | --- | --- | --- | --- | --- | --- |
| Attribute | Mean^1^ | SE^2^ | SD | SE |  | Attribute | Mean^1^ | | | SE^2^ | SD | | | | SE | | |
| Non-HIV services available at the same consultation | 2.35 | 0.12** | 2.07 | 0.12** |  | Non-HIV services available at the same consultation | | 1.00 | 0.09** | | | 1.26 | 0.08** | | |  |  |
| Providers are respectful and welcoming | 1.80 | 0.09** | 1.51 | 0.09** |  | Providers are respectful and pleasant | | 1.39 | 0.10** | | | 1.37 | 0.08** | | |  |  |
| Mother support groups available | 1.02 | 0.07** | 0.66 | 0.10** |  | Providers involve husband/family in care | | 0.76 | 0.07** | | | 0.90 | 0.08** | | |  |  |
| Counseling services available | 0.94 | 0.07** | -0.57 | 0.14** |  | Counseling services available | | 0.59 | 0.06** | | | 0.75 | 0.06** | | |  |  |
|  |  |  |  |  |  | Health center (vs. mobile clinic) | | 0.13 | 0.08* | | | -0.24 | 0.19 | | |  |  |
| Hospital (vs. health center) | 0.36 | 0.06** | 0.88 | 0.09** |  | Hospital (vs. mobile clinic) | | 0.18 | 0.07** | | | 0.36 | 0.15** | | |  |  |
| Cost (continuous in 100 Birr)^3^ | -0.47 | 0.04** |  |  |  | Cost (continuous in 100 MTn)^3^ | | -0.17 | 0.03** | | |  | |  | | |  |
|  |  |  |  |  |  |  | |  |  | | |  | |  | | |  |
| Pregnant × Non-HIV services available | -0.31 | 0.27 |  |  |  | Pregnant × Non-HIV services available | | 0.12 | 0.12 | | |  | |  | | |  |
| Pregnant × Providers are respectful | -0.13 | 0.21 |  |  |  | Pregnant × Providers are respectful | | 0.42 | 0.13** | | |  | |  | | |  |
| Pregnant × Mother support groups available | 0.34 | 0.17** |  |  |  | Pregnant × Providers involve husband/family | | -0.18 | 0.10* | | |  | |  | | |  |
| Pregnant × Counseling services available | 0.03 | 0.19 |  |  |  | Pregnant × Counseling services available | | -0.04 | 0.09 | | |  | |  | | |  |
|  |  |  |  |  |  | Pregnant × Health center (vs. mobile clinic) | | 0.06 | 0.11 | | |  | |  | | |  |
| Pregnant × Hospital (vs. health center) | 0.05 | 0.18 |  |  |  | Pregnant × Hospital (vs. mobile clinic) | | -0.06 | 0.11 | | |  | |  | | |  |
| Pregnant × Cost (continuous in 100 Birr)^3^ | 0.10 | 0.09 |  |  |  | Pregnant × Cost (continuous in 100 MTn)^3^ | | -0.05 | 0.04 | | |  | |  | | |  |
|  | | | | |  |  | | | | | | | | | | | |
| Model diagnostics | | | | |  | Model diagnostics | | | | | | | | | | | |
| Number of respondents | 1,013 | | | |  | Number of respondents | 1,020 | | | | | | | | | | |
| Number of observations | 16,192 | | | |  | Number of observations | 16,156 | | | | | | | | | | |
| Log-likelihood | -3561.1 | | | |  | Log-likelihood | -4175.0 | | | | | | | | | | |
| Likelihood ratio χ2 | 935.87 | | | |  | Likelihood ratio χ2 | 625.20 | | | | | | | | | | |

^1^ Mean β coefficients show estimated utility of each attribute, where positive coefficients indicate positive preference. Positive coefficients for pregnant × <attribute> interaction terms indicate that pregnant women place higher preference on that attribute than do non-pregnant women. The overall preference for a service scenario is calculated as the sum of the products of the main effects and the interaction terms.

^2^ **p* < .05, ***p* < .01

^3^ Currency equivalents in USD are 100 Ethiopian Birr = 5.12 USD and 100 Mozambican MTn = 3.20 USD, using period average exchange rates for the dates of data collection, extracted from OANDA.com (Ethiopia: 16 Apr 2014 to 12 Jun 2014; Mozambique: 8 Apr 2014 to 23 May 2014).
