# Supplementary material for: How Can the Health System Retain Women in HIV Treatment for a Lifetime? A Discrete Choice Experiment in Ethiopia and Mozambique
Source: PLoS One. 2016 Aug 23;11(8):e0160764. doi: 10.1371/journal.pone.0160764 (PMC4994936; doi:10.1371/journal.pone.0160764)
Supplement: S2 Table — (DOCX) [file pone.0160764.s005.docx]

S2 Table. Results of mixed logit regression models with interaction terms with ART status (Not currently on ART vs. on ART)

| Ethiopia | | | | |  | Mozambique | | | | |
| --- | --- | --- | --- | --- | --- | --- | --- | --- | --- | --- |
| Attribute | Mean^1^ | SE^2^ | SD | SE |  | Attribute | Mean^1^ | SE^2^ | SD | SE |
| Non-HIV services available at the same consultation | 2.19 | 0.39** | 2.08 | 0.12** |  | Non-HIV services available at the same consultation | 0.69 | 0.16** | 1.26 | 0.08** |
| Providers are respectful and welcoming | 1.53 | 0.31** | 1.51 | 0.09** |  | Providers are respectful and pleasant | 1.82 | 0.18** | 1.39 | 0.09** |
| Mother support groups available | 1.29 | 0.26** | 0.67 | 0.10** |  | Providers involve husband/partner in care | 0.52 | 0.14** | 0.90 | 0.08** |
| Counseling services available | 1.43 | 0.28** | -0.59 | 0.14** |  | Counseling services available | 0.53 | 0.12** | 0.74 | 0.06** |
|  |  |  |  |  |  | Health center (vs. mobile clinic) | 0.17 | 0.15 | 0.18 | 0.22 |
| Hospital (vs. health center) | 0.29 | 0.25 | 0.87 | 0.09** |  | Hospital (vs. mobile clinic) | 0.40 | 0.15** | 0.40 | 0.14** |
| Cost (continuous in 100 Birr)^3^ | -0.55 | 0.14** |  |  |  | Cost (continuous in 100 MTn)^3^ | -0.08 | 0.05 |  |  |
|  |  |  |  |  |  |  |  |  |  |  |
| Not on ART × Non-HIV services available | -0.11 | 0.39 |  |  |  | Not on ART × Non-HIV services available | -0.42 | 0.18* |  |  |
| Not on ART × Providers are respectful | -0.27 | 0.31 |  |  |  | Not on ART × Providers are respectful | 0.26 | 0.18 |  |  |
| Not on ART × Mother support groups available | 0.25 | 0.26 |  |  |  | Not on ART × Providers involve husband/family | -0.18 | 0.15 |  |  |
| Not on ART × Counseling services available | 0.51 | 0.28 |  |  |  | Not on ART × Counseling services available | -0.05 | 0.12 |  |  |
|  | -0.08 | 0.26 |  |  |  | Not on ART × Health center (vs. mobile clinic) | 0.02 | 0.16 |  |  |
| Not on ART × Hospital (vs. health center) | -0.09 | 0.15 |  |  |  | Not on ART × Hospital (vs. mobile clinic) | 0.29 | 0.16 |  |  |
| Not on ART × Cost (continuous in 100 Birr)^3^ | -0.09 | 0.15 |  |  |  | Not on ART × Cost (continuous in 100 MTn)^3^ | 0.12 | 0.06* |  |  |
|  | | | | |  |  | | | | |
| Model diagnostics | | | | |  | Model diagnostics | | | | |
| Number of respondents | 1,013 | | | |  | Number of respondents | 1,020 | | | |
| Number of observations | 16,176 | | | |  | Number of observations | 16,124 | | | |
| Log-likelihood | -3559.3 | | | |  | Log-likelihood | -4162.6 | | | |
| Likelihood ratio χ2 | 933.67 | | | |  | Likelihood ratio χ2 | 627.61 | | | |

^1^ Mean β coefficients show estimated utility of each attribute, where positive coefficients indicate positive preference. Positive coefficients for Not currently on ART × <attribute> interaction terms indicate that women not currently on ART place higher preference on that attribute than do women currently on ART. The overall preference for a service scenario is calculated as the sum of the products of the main effects and the interaction terms.

^2^ **p* < .05, ***p* < .01

^3^ Currency equivalents in USD are 100 Ethiopian Birr = 5.12 USD and 100 Mozambican MTn = 3.20 USD, using period average exchange rates for the dates of data collection, extracted from OANDA.com (Ethiopia: 16 Apr 2014 to 12 Jun 2014; Mozambique: 8 Apr 2014 to 23 May 2014).
