# Supplementary material for: How Can the Health System Retain Women in HIV Treatment for a Lifetime? A Discrete Choice Experiment in Ethiopia and Mozambique
Source: PLoS One. 2016 Aug 23;11(8):e0160764. doi: 10.1371/journal.pone.0160764 (PMC4994936; doi:10.1371/journal.pone.0160764)
Supplement: S3 Table — (DOCX) [file pone.0160764.s006.docx]

S3 Table. Results of mixed logit regression models with interaction terms with current potential B+ clients (women pregnant or breastfeeding and not on ART or on ART during pregnancies only (pMTCT) vs. others)

| Ethiopia | | | | |  | Mozambique | | | | |
| --- | --- | --- | --- | --- | --- | --- | --- | --- | --- | --- |
| Attribute | Mean^1^ | SE^2^ | SD | SE |  | Attribute | Mean^1^ | SE^2^ | SD | SE |
| Non-HIV services available at the same consultation | 2.31 | 0.12** | 2.06 | 0.12** |  | Non-HIV services available at the same consultation | 0.94 | 0.08** | 1.25 | 0.08** |
| Providers are respectful and welcoming | 1.80 | 0.09** | 1.49 | 0.09** |  | Providers are respectful and pleasant | 1.48 | 0.09** | 1.37 | 0.08** |
| Mother support groups available | 1.01 | 0.06** | -0.64 | 0.10** |  | Providers involve husband/partner in care | 0.73 | 0.07** | 0.89 | 0.08** |
| Counseling services available | 0.94 | 0.07** | -0.54 | 0.15** |  | Counseling services available | 0.58 | 0.06** | 0.75 | 0.06** |
|  |  |  |  |  |  | Health center (vs. mobile clinic) | 0.16 | 0.07* | -0.24 | 0.19 |
| Hospital (vs. health center) | 0.34 | 0.06** | 0.86 | 0.09** |  | Hospital (vs. mobile clinic) | 0.16 | 0.07* | 0.37 | 0.15* |
| Cost (continuous in 100 Birr)^3^ | -0.45 | 0.03** |  |  |  | Cost (continuous in 100 MTn)^3^ | -0.18 | 0.03** |  |  |
|  |  |  |  |  |  |  |  |  |  |  |
| B+ client × Non-HIV services available | -0.38 | 0.38 |  |  |  | B+ client × Non-HIV services available | 0.32 | 0.13* |  |  |
| B+ client × Providers are respectful | -0.50 | 0.30 |  |  |  | B+ client × Providers are respectful | 0.34 | 0.13* |  |  |
| B+ client × Mother support groups available | 0.66 | 0.24** |  |  |  | B+ client × Providers involve husband/family | -0.18 | 0.11 |  |  |
| B+ client × Counseling services available | -0.05 | 0.25 |  |  |  | B+ client × Counseling services available | -0.03 | 0.09 |  |  |
|  |  |  |  |  |  | B+ client × Health center (vs. mobile clinic) | 0.03 | 0.12 |  |  |
| B+ client × Hospital (vs. health center) | 0.50 | 0.25* |  |  |  | B+ client × Hospital (vs. mobile clinic) | -0.03 | 0.11 |  |  |
| B+ client × Cost (continuous in 100 Birr)^3^ | -0.13 | 0.13 |  |  |  | B+ client × Cost (continuous in 100 MTn)^3^ | -0.02 | 0.04 |  |  |
|  | | | | |  |  | | | | |
| Model diagnostics | | | | |  | Model diagnostics | | | | |
| Number of respondents | 1,013 | | | |  | Number of respondents | 1,020 | | | |
| Number of observations | 16,192 | | | |  | Number of observations | 16,156 | | | |
| Log-likelihood | -3556.7 | | | |  | Log-likelihood | -4175.1 | | | |
| Likelihood ratio χ2 | 932.18 | | | |  | Likelihood ratio χ2 | 619.54 | | | |

^1^ Mean β coefficients show estimated utility of each attribute, where positive coefficients indicate positive preference. Positive coefficients for B+ client × <attribute> interaction terms indicate that current potential B+ clients (women who are pregnant or breastfeeding and not on ART or on ART during pregnancies only (pMTCT)) place higher preference on that attribute than do women currently on ART. The overall preference for a service scenario is calculated as the sum of the products of the main effects and the interaction terms.

^2^ **p* < .05, ***p* < .01

^3^ Currency equivalents in USD are 100 Ethiopian Birr = 5.12 USD and 100 Mozambican MTn = 3.20 USD, using period average exchange rates for the dates of data collection, extracted from OANDA.com (Ethiopia: 16 Apr 2014 to 12 Jun 2014; Mozambique: 8 Apr 2014 to 23 May 2014).
