# Supplementary material for: How Can the Health System Retain Women in HIV Treatment for a Lifetime? A Discrete Choice Experiment in Ethiopia and Mozambique
Source: PLoS One. 2016 Aug 23;11(8):e0160764. doi: 10.1371/journal.pone.0160764 (PMC4994936; doi:10.1371/journal.pone.0160764)
Supplement: S4 Table — (DOCX) [file pone.0160764.s007.docx]

S4 Table. Results of mixed logit regression models with interaction terms with age less than 25 (<25 vs. 25+ years old)

| Ethiopia | | | | |  | Mozambique | | | | |
| --- | --- | --- | --- | --- | --- | --- | --- | --- | --- | --- |
| Attribute | Mean^1^ | SE^2^ | SD | SE |  | Attribute | Mean^1^ | SE^2^ | SD | SE |
| Non-HIV services available at the same consultation | 2.34 | 0.12** | 2.08 | 0.12** |  | Non-HIV services available at the same consultation | 1.04 | 0.09** | 1.28 | 0.08** |
| Providers are respectful and welcoming | 1.80 | 0.09** | 1.51 | 0.09** |  | Providers are respectful and pleasant | 1.54 | 0.10** | 1.39 | 0.08** |
| Mother support groups available | 1.04 | 0.07** | 0.65 | 0.10** |  | Providers involve husband/partner in care | 0.71 | 0.08** | 0.91 | 0.08** |
| Counseling services available | 0.91 | 0.72** | -0.56 | 0.14** |  | Counseling services available | 0.64 | 0.06** | 0.76 | 0.06** |
|  |  |  |  |  |  | Health center (vs. mobile clinic) | 0.09 | 0.08* | -0.25 | 0.18 |
| Hospital (vs. health center) | 0.36 | 0.06** | 0.88 | 0.09** |  | Hospital (vs. mobile clinic) | 0.16 | 0.08 | 0.38 | 0.14** |
| Cost (continuous in 100 Birr)^3^ | -0.46 | 0.03** |  |  |  | Cost (continuous in 100 MTn)^3^ | -0.15 | 0.03** |  |  |
|  |  |  |  |  |  |  |  |  |  |  |
| Age <25 years × Non-HIV services available | -0.28 | 0.29 |  |  |  | Age <25 years × Non-HIV services available | 0.04 | 0.13 |  |  |
| Age <25 years × Providers are respectful | -0.17 | 0.23 |  |  |  | Age <25 years × Providers are respectful | 0.14 | 0.13 |  |  |
| Age <25 years × Mother support groups available | 0.12 | 0.19 |  |  |  | Age <25 years × Providers involve husband/family | -0.08 | 0.10 |  |  |
| Age <25 years × Counseling services available | 0.19 | 0.21 |  |  |  | Age <25 years × Counseling services available | -0.14 | 0.09 |  |  |
|  |  |  |  |  |  | Age <25 years × Health center (vs. mobile clinic) | 0.15 | 0.11 |  |  |
| Age <25 years × Hospital (vs. health center) | 0.06 | 0.20 |  |  |  | Age <25 years × Hospital (vs. mobile clinic) | -0.00 | 0.11 |  |  |
| Age <25 years × Cost (continuous in 100 Birr)^3^ | -0.01 | 0.10 |  |  |  | Age <25 years × Cost (continuous in 100 MTn)^3^ | -0.07 | 0.04 |  |  |
|  | | | | |  |  | | | | |
| Model diagnostics | | | | |  | Model diagnostics | | | | |
| Number of respondents | 1,013 | | | |  | Number of respondents | 1,020 | | | |
| Number of observations | 16,192 | | | |  | Number of observations | 16,156 | | | |
| Log-likelihood | -3563.7 | | | |  | Log-likelihood | -4179.6 | | | |
| Likelihood ratio χ2 | 936.85 | | | |  | Likelihood ratio χ2 | 636.72 | | | |

^1^ Mean β coefficients show estimated utility of each attribute, where positive coefficients indicate positive preference. Positive coefficients for age <25 years × <attribute> interaction terms indicate that women under 25 years place higher preference on that attribute than do women at least 25 years old. The overall preference for a service scenario is calculated as the sum of the products of the main effects and the interaction terms.

^2^ **p* < .05, ***p* < .01

^3^ Currency equivalents in USD are 100 Ethiopian Birr = 5.12 USD and 100 Mozambican MTn = 3.20 USD, using period average exchange rates for the dates of data collection, extracted from OANDA.com (Ethiopia: 16 Apr 2014 to 12 Jun 2014; Mozambique: 8 Apr 2014 to 23 May 2014).
